# Beam‐axis stability during gantry rotation measured with scintillation dosimetry

**DOI:** 10.1002/acm2.70594

**Published:** 2026-05-14

**Authors:** Shingo Ohira, Takeshi Ohta, Honoka Sugaya, Tenyoh Suzuki, Yuki Nozawa, Masanari Minamitani, Atsuto Katano, Takuya Hayashi, Hideomi Yamashita, Keiichi Nakagawa

**Affiliations:** ^1^ Department of Radiological Sciences Graduate School of Human Health Sciences Tokyo Metropolitan University Arakawa‐ku Tokyo Japan; ^2^ Department of Comprehensive Radiation Oncology Graduate School of Medicine The University of Tokyo Bunkyo‐ku Tokyo Japan; ^3^ Department of Radiology The University of Tokyo Hospital Bunkyo‐ku Tokyo Japan

**Keywords:** beam‐axis, scintillation, stability

## Abstract

**Purpose:**

Volumetric modulated arc therapy (VMAT) requires high mechanical precision due to continuous gantry rotation. This study aims to investigate beam‐axis stability during VMAT delivery and evaluate scintillation‐based imaging as a complementary quality assurance (QA) tool.

**Methods:**

Beam‐axis deviations were measured using a scintillation plate dosimeter, acquiring images at 5 frames per second in axial and coronal planes. Nine conformal arc beams with a field size of 1 × 1 cm^2^ were delivered to a phantom under three configurations of the electric portal imaging device (EPID) and x‐ray volume imaging (XVI) systems: middle, retracted, and extended. Short‐ and long‐term reproducibility was assessed through repeated measurements. Deviations between the radiation dose center and the beam axis were analyzed in X, Y, and Z directions, and three‐dimensional (3D) displacements were calculated.

**Results:**

Short‐term reproducibility showed maximum standard deviations (SDs) of 0.05, 0.06, and 0.11 mm for 4 MV, 6 MV, and 6 FFF beams, respectively. Long‐term reproducibility exhibited maximum SDs of 0.15, 0.13, and 0.19 mm, respectively. Angle‐dependent variations were most prominent at gantry angles of 0° and ±180°, consistent with gantry sag under gravity. The 3D displacements between the radiation dose center and the beam axis remained below 1 mm across all energies: the average (maximum) displacement ranged from 0.47 mm (0.96 mm) to 0.52 mm (0.97 mm) for 4 MV, 0.41 mm (0.68 mm) to 0.44 mm (0.76 mm) for 6 MV, and 0.42 mm (0.88 mm) to 0.47 mm (0.95 mm) for 6FFF. No relevant differences were observed among the three EPID/XVI configurations, indicating that these devices have a minimal impact on gantry mechanical stability.

**Conclusions:**

Scintillation‐based imaging enables real‐time, high‐resolution evaluation of beam‐axis stability during VMAT. Deviations were consistently within submillimeter accuracy, supporting its use as a complementary QA method to conventional tests.

## 1 INTRODUCTION

Volumetric modulated arc therapy (VMAT) is widely adopted in clinical radiotherapy because it allows efficient delivery of highly conformal dose distributions for targets while minimizing doses for organs at risk.^1^, ^2^ In VMAT, the linear accelerator gantry rotates continuously while multileaf collimator (MLC) patterns and the dose rate are varied dynamically.^3^ This simultaneous motion requires very high mechanical precision to keep the beam accurately aligned with the treatment isocenter. Even small deviations of the beam axis during gantry rotation can affect treatment accuracy. This is especially important in cranial stereotactic irradiation (STI), where submillimeter precision is necessary to ensure effective dose delivery and prevent damage to surrounding healthy tissues.^4^, ^5^, ^6^

Quality assurance (QA) is essential for ensuring that the radiation beam remains stable during rotational treatments. The star‐shot test is one of the most common approaches for evaluating beam‐axis alignment.^7^ In electronic portal imaging device (EPID)‐based star‐shot testing, static radiation beams are delivered from several gantry angles and recorded on the EPID.^8^ The intersection of the beams’ central axes is then analyzed to assess the accuracy of the treatment isocenter. Although the EPID‐based test is widely used, several limitations remain. This method evaluates static beams and therefore cannot capture the stability of the beam axis during continuous gantry rotation.^9^ Moreover, EPID images are acquired on the detector plane rather than at the isocenter, requiring geometric corrections that may introduce errors if not applied properly. In addition, the EPID itself may shift slightly depending on the gantry angle.^10^

Recent advances in scintillation dosimeters and high‐frame‐rate imaging have provided new opportunities for more detailed QA. Newly developed scintillation‐based dosimetry enables real‐time, two‐dimensional imaging of radiation fields with high spatial resolution.^11^ By acquiring hundreds of images during gantry rotation, subtle angle‐dependent variations in beam‐axis position can be identified. By performing measurements in the axial plane, the beam‐axis position in the X (left‐right) and Y (anterior‐posterior) directions during gantry rotation can be precisely monitored, while measurements in the coronal plane allow assessment in the Z direction (superior‐inferior). Combining these measurements provides the potential to evaluate the three‐dimensional (3D) stability of the beam axis, offering an additional understanding of mechanical stability.

The purpose of this study was to investigate beam‐axis stability during rotational irradiation using a scintillation plate dosimeter and to evaluate its potential as an additional, complementary QA method alongside conventional tests such as the EPID‐based star‐shot, particularly for high‐precision treatments

## 2 MATERIALS AND METHODS

As this study was conducted using a phantom, ethical approval was not necessary.

### 2.1 Scintillation plate dosimeter

**FIGURE 1:**
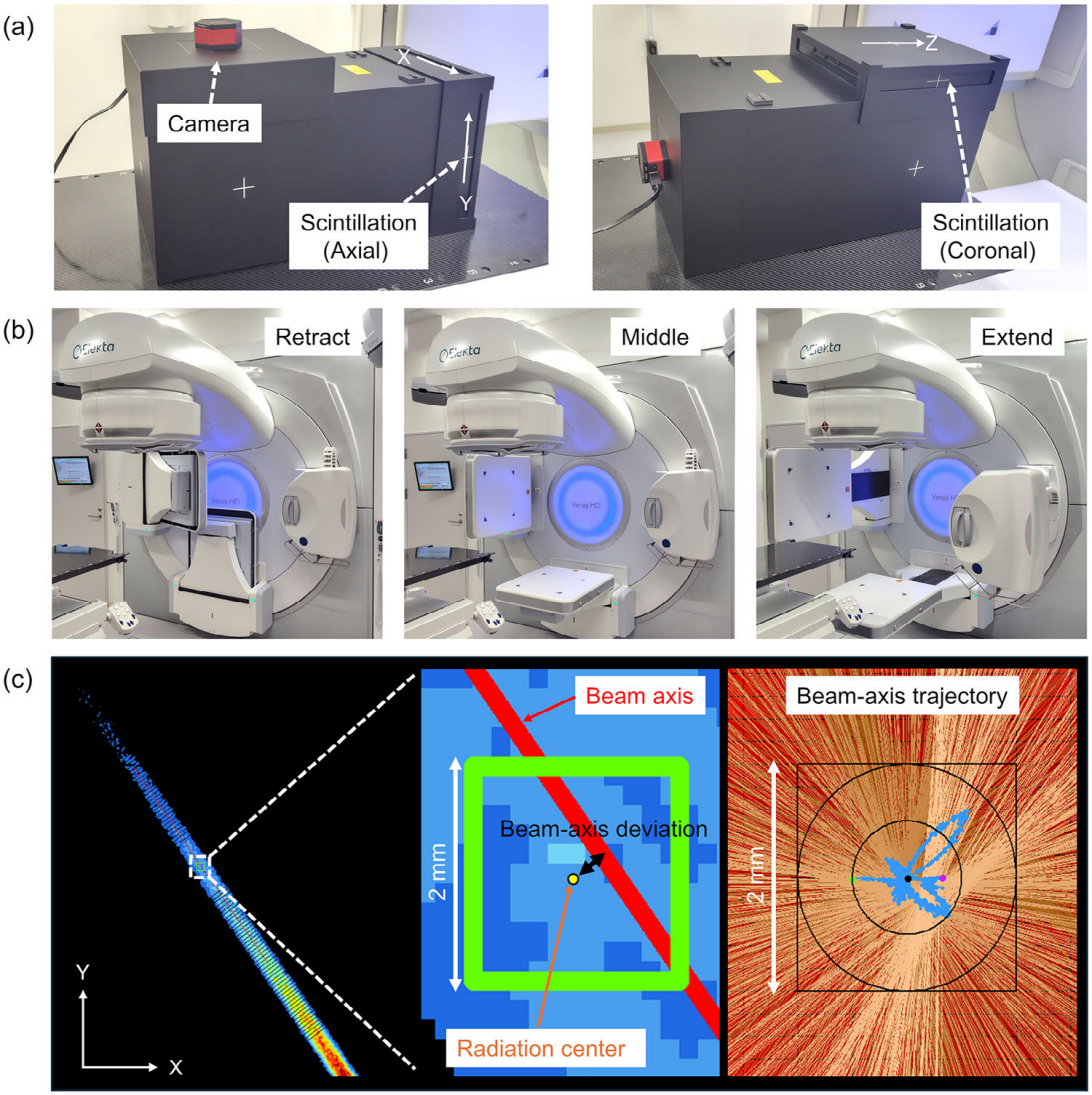
Experimental setup and measurement procedure. (a) Schematic of the scintillation plate dosimeter positioned at the isocenter. (b) Three configurations of the electric portal imaging device (EPID) and x‐ray volume imaging (XVI) systems were investigated: Retract, Middle, and Extend. (c) Illustration of beam‐axis deviation analysis. The centroid of regions receiving more than 20% of the dose was defined as the radiation center. For each image during rotation irradiation, 50 concentric profiles were generated around the radiation center, and the line passing through the centroids of these profiles was defined as the beam axis. Beam‐axis deviations were calculated as the shortest distance from the radiation center to the beam axis.

In this study, a scintillation plate dosimeter (DoseScope, Chiyoda Technol Corp., Tokyo, Japan) was used for measurements.^11^ The plastic scintillator (240 × 240 × 2 mm^3^), composed of polystyrene with 1% diphenyloxazole and 0.08% red wavelength shifter (610 nm), has a physical density of 1.05 g/cm^3^ and an electron density of 1.02. It was placed between two 2 cm PMMA buildup layers, giving a total thickness of 4.2 cm. Orientation changes of the scintillation plate dosimeter enable measurements in both the axial and coronal planes (Figure 1a). The scintillation light generated by radiation exposure is reflected at a 45° angle by the mirror. A CMOS planetary RGB camera (Uranus‐C, Player One Astronomy, Suzhou, China) with a Sony IMX585 sensor (dark noise 0.7e^−^, read noise 1e^−^) was used to detect scintillation light. The image was configured to 2000 × 2000 pixels, with a 650 × 650 pixel area covering the scintillator plate (pixel size: 0.37 × 0.37 mm^2^).

### 2.2 Beam delivery

The scintillation plate dosimeter was scanned using a computed tomography (CT) (SOMATOM go. Sim, Siemens Healthineers, Germany) with a slice thickness of 1 mm in both the axial and coronal planes, and the CT images were transferred to a treatment planning system (Monaco, Elekta AB, Stockholm, Sweden). The outline of the plastic scintillator was contoured, and a conformal arc beam was generated at the center of the plastic scintillator. Table 1 shows the beam delivery parameters, and nine different arc beams were used in this study (VersaHD, Elekta AB). To avoid MLC positional variation due to gravity, which depends on the gantry angle,^12^ the collimator was rotated 90° under all conditions. This simplified geometry ensures that any observed deviations are primarily attributable to the gantry's mechanical characteristics, such as gravitational sag, rather than leaf‐sequencing errors. No upper limit was imposed on the dose rate during irradiation of 1000 MU. For the 6 FFF beam, a dose rate of 350 MU/min was used, representing approximately half value without any dose rate limitations. In clinical practice, imaging devices such as the EPID or x‐ray volume imaging (XVI) system (kV x‐ray tube and kV flat panel) mounted on a linear accelerator are frequently extended or retracted depending on the treatment procedure. These positional changes alter the mechanical balance of the gantry, which may in turn influence the stability and alignment of the beam axis. In this study, three distinct configurations were investigated (Figure 1b): the EPID/XVI systems were in the retracted, middle, and extended positions.

**TABLE 1:** Beam delivery parameters.

Protocol number #	Beam energy	MU	Field size (cm^2^)	Collimator angle (°)	Dose rate (MU/min)	Gantry rotation	Imager position
1	4X	1000	1 × 1	90	290	CCW, CW	Middle
2							Retract
3							Extend
4	6X				500		Middle
5							Retract
6							Extend
7	6FFF				690		Middle
8							Retract
9							Extend
10					350		Middle

The center of the scintillation plate dosimeter was placed at the isocenter in the same orientation as during the CT simulation, and all beams were delivered in both the axial and coronal planes. To evaluate the short‐term reproducibility of the beam‐axis stability, measurements for protocol numbers 1, 4, and 7 were repeated five times on the same day. For long‐term reproducibility, the same measurements were repeated weekly. To compare the influence of EPID/XVI systems on beam‐axis stability, each protocol (#1–9) was measured once, and the average values of CCW and CW directions were compared.

### 2.3 Data analysis

The CMOS camera acquired scintillation light images during the dose delivery at 5 frames per second (FPS), and approximately 400–1000 images were obtained depending on the beam delivery conditions. The application software was developed in LabVIEW (National Instruments, Austin, USA). Vignetting correction and 3 × 3 median filtering for spike noise (caused by scattered rays entering the sensor element) reduction were applied in real time during imaging.^11^ After dose delivery was completed, the images were accumulated and converted into a dose distribution. Figure 1c shows the calculation of beam‐axis deviations from the center of the dose distribution. Regions with more than 20% of the dose were masked, and their centroid was defined as the radiation center. For each image, 50 concentric profiles were generated around the radiation center relative to the beam axis, and the line passing through the centroids of these profiles was defined as the beam axis. The deviation of the beam axis was defined as the shortest distance from the radiation center to the beam axis. The first and last images were assigned gantry angles of 180° or −180°, respectively, and the gantry angles for all intermediate images were determined by linear interpolation. Subsequently, the beam‐axis deviations were resampled at 1° intervals of gantry angle. For X and Y directions, the beam‐axis deviations were determined based on the axial plane images, and the Z direction was coronal plane images. For the 3D displacement, the square root of the sum of squares of the X, Y, and Z components was calculated for comparison.

## 3 RESULTS

**FIGURE 2:**
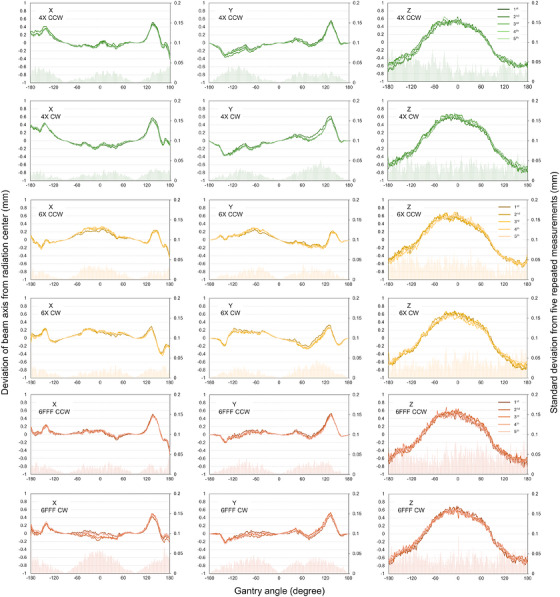
Short‐term reproducibility of beam‐axis deviations from radiation center during rotational irradiation (protocol #1).

Figure 2 shows the short‐term reproducibility of the beam‐axis stability during rotational irradiation (protocol #1, 4, and 7). In the X and Y directions, slight differences in the beam‐axis stability were observed depending on the gantry rotation direction (CCW vs. CW). Moreover, the magnitude of the displacement between the radiation center and the beam axis varied with beam energy. The short‐term reproducibility of the beam‐axis stability was high at all gantry angles, with maximum standard deviations (SDs) across five measurements of 0.05, 0.06, and 0.11 mm for 4 MV, 6 MV, and 6 FFF, respectively. The deviations between the radiation center and the beam axis ranged from −0.41 to 0.52 mm, −0.46 to 0.33 mm, and −0.48 to 0.53 mm for 4 MV, 6 MV, and 6 FFF, respectively. In the Z direction as well, short‐term reproducibility was high, with a maximum SD of 0.1 mm for all energies. The displacement between the radiation center and the beam axis was greatest at gantry angles of 0° or 180° (−180°), ranging from −0.76 to 0.65 mm, −0.69 to 0.70 mm, and −0.85 to 0.68 mm for 4 MV, 6 MV, and 6 FFF, respectively.

**FIGURE 3:**
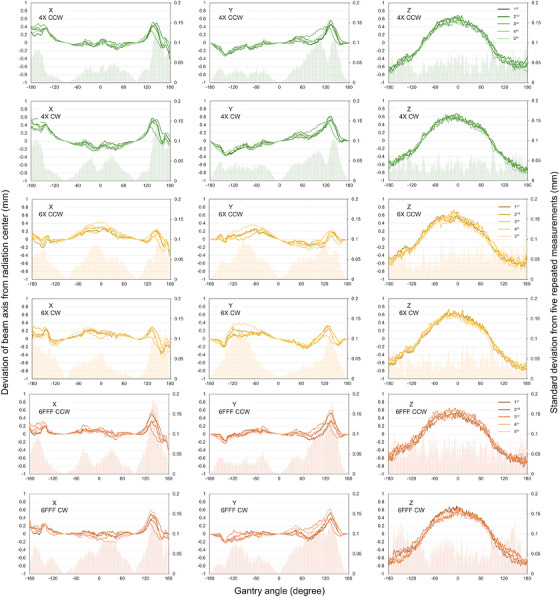
Long‐term reproducibility of beam‐axis deviations from radiation center during rotational irradiation (protocol #1).

Figure 3 shows the long‐term (weekly) reproducibility of the beam‐axis stability during rotational irradiation (protocol #1, 4, and 7). The variation among the long‐term five measurements was higher than the short‐term, and the maximum SD for 4 MV was 0.15, 0.11, and 0.13 mm in the X, Y, and Z directions, respectively. For 6 MV, these were 0.12, 0.13, and 0.11 mm in the X, Y, and Z directions, respectively, and 0.19, 0.18, and 0.14 mm for 6FFF.

**FIGURE 4:**
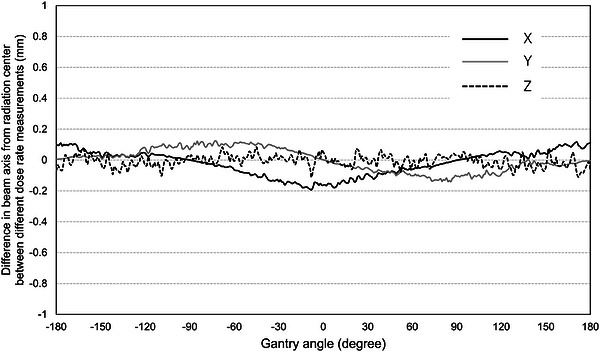
Difference in the beam axis from the radiation center, measured using two different beam delivery parameters (protocol numbers 7 and 10 in Table 1).

The difference in the beam axis from the radiation center, measured using two different beam delivery parameters (protocol numbers 7 and 10 in Table 1), is shown in Figure 4. The mean difference between two beam delivery parameters (dose rate) was −0.02, −0.001, and −0.007 mm in the X, Y, and Z directions, respectively. The maximum difference of −0.19 mm was observed in the X direction.

**FIGURE 5:**
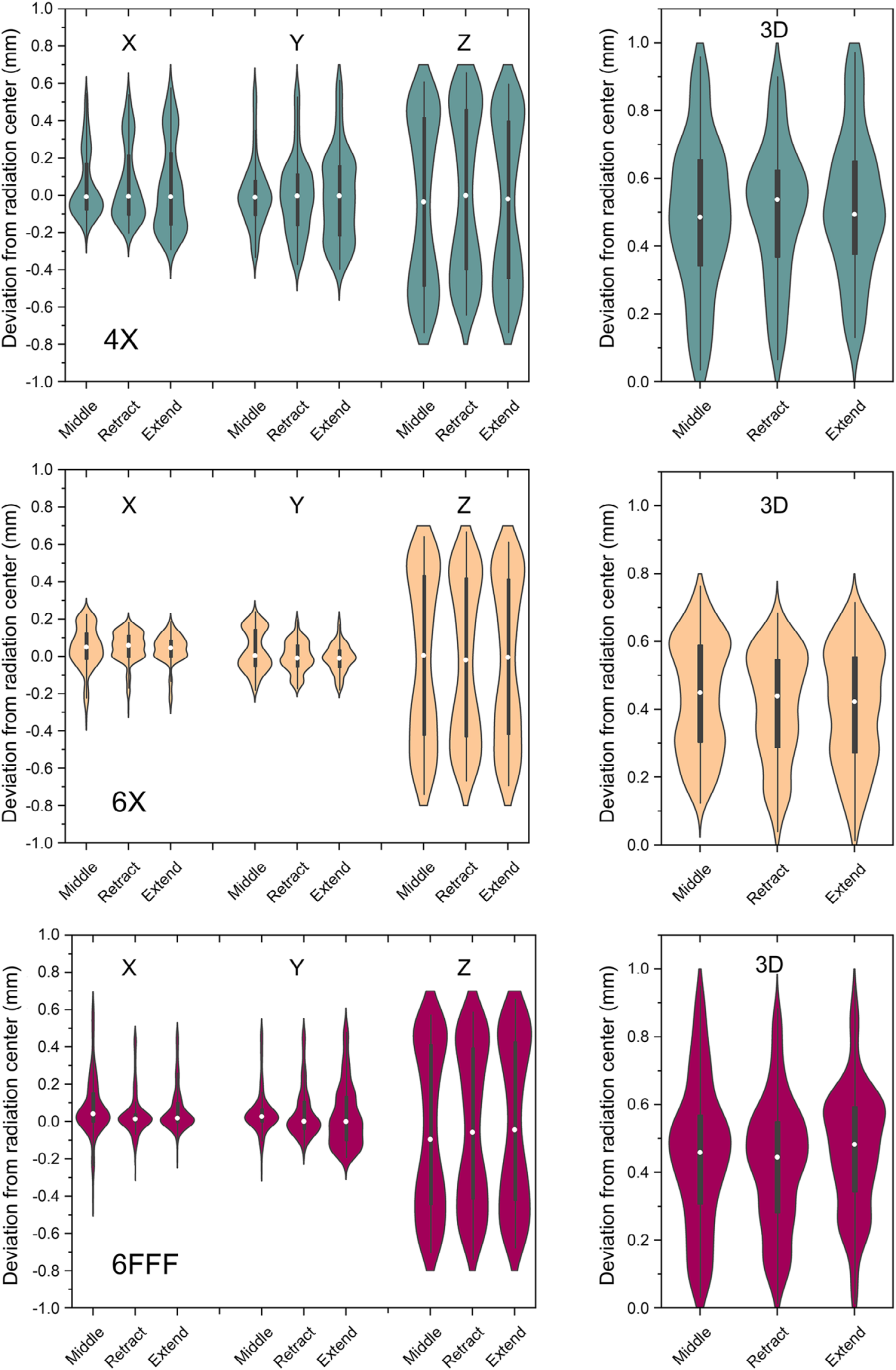
Comparison of 3D beam‐axis deviations among different electric portal imaging device (EPID)/x‐ray volume imaging (XVI) configurations.

Figure 5 shows the comparison of the deviations between the radiation center and the beam axis among three distinct configurations of EPID and XVI systems. Across all photon energies and directions (X, Y, and Z directions), there were no clear differences in the displacement between the radiation center and the beam axis among the Middle, Retract, and Extend positions. The 3D displacement was consistently small across all energy levels (4 MV, 6 MV, and 6FFF) and positional conditions (Middle, Retract, and Extend). The average (maximum) 3D displacement ranged from 0.47 mm (0.96 mm) to 0.52 mm (0.97 mm) for 4 MV, 0.41 mm (0.68 mm) to 0.44 mm (0.76 mm) for 6 MV, and 0.42 mm (0.88 mm) to 0.47 mm (0.95 mm) for 6FFF.

## 4 DISCUSSIONS

The present study investigated the stability of the beam axis during rotational irradiation using a scintillation plate dosimeter with sub‐millimeter spatial accuracy and sub‐second temporal resolution. Our findings demonstrate that while long‐term reproducibility exhibits slightly more variation than short‐term measurements—likely due to minor setup uncertainties during weekly dosimeter placement—the overall stability remains highly robust. Furthermore, the positioning of EPID and XVI accessories did not significantly affect gantry mechanical stability, suggesting that these components are well‐balanced within the linear accelerator's design.

In recent years, there has been a clear shift from fixed‐field IMRT to rotational techniques, with VMAT becoming the predominant treatment approach due to its efficiency and superior dose conformity.^13^, ^14^ Since VMAT relies on stable beam trajectories during gantry rotation, the high short‐ and long‐term reproducibility demonstrated in this study provides essential evidence for ensuring treatment accuracy. Du et al. investigated the gantry sag using EPID with the fixed radiation fields at 10° gantry angle intervals, and found that the maximum gantry sag varied from 0.7 to 1.0 mm.^12^ Evaluating the gantry sag using an EPID is challenging, as it is difficult to distinguish whether the observed sag originates from the EPID itself or from the gantry.^15^ The film is typically placed stationary on the treatment couch, and thus, it is entirely independent of mechanical displacement of the imaging panel itself.^7^ However, film acts as an integrating detector, which poses a limitation in evaluating gantry sag during continuous rotation.^12^ In contrast, our scintillation‐based method enables continuous tracking of the beam axis throughout the gantry rotation. The observation that Z‐direction deviations peaked at 0° or ± 180° aligns with the physical impact of gravity on gantry sag, confirming the sensitivity of our approach. Tsuneda et al. utilized a combination of a column‐shaped plastic scintillator and a cone‐shaped mirror to estimate gantry positional deviations through a specific optical projection.^16^ In contrast, our method employs a planar (plate‐shaped) plastic scintillator, which enables the direct and straightforward measurement of the beam axis in the plane of interest. Furthermore, since our system is based on a commercially available dosimeter widely used for VMAT verification in Japan, the proposed QA procedure for beam‐axis stability can be easily integrated into routine clinical practice without the need for specialized experimental setups.

From a clinical perspective, the observed 3D deviations (maximum < 1 mm) fall well within the tolerances recommended for stereotactic radiosurgery and stereotactic body radiotherapy.^17^, ^18^ In cranial STI, precise dose delivery is required to maximize tumor control while minimizing exposure to surrounding healthy brain tissue. Commonly, very tight planning target volume margins are employed, often on the order of 1–2 mm, to account for setup uncertainties and mechanical deviations.^19^, ^20^ Therefore, rigorous verification of mechanical accuracy and continuous monitoring of beam alignment are particularly essential in cranial STI. In this context, while conventional QA methods such as Winston–Lutz and star‐shot tests remain valuable for assessing isocenter accuracy,^21^ the scintillation‐based approach presented in this study provides complementary information by enabling real‐time, 3D evaluation of beam‐axis stability throughout gantry rotation. This additional capability allows the detection of subtle, angle‐dependent deviations that might not be captured by conventional QA methods, thereby improving the overall QA of high‐precision cranial treatments.

Several limitations warrant consideration. First, we used a static 1 × 1 cm^2^ field under controlled conditions to isolate mechanical gantry stability. However, in clinical VMAT, the MLC leaves move dynamically and the dose rate fluctuates depending on the patients. Second, this study evaluated only a single linear accelerator model, and it is uncertain whether the findings can be generalized to other treatment units. Moreover, even among machines of the same model, differences in mechanical characteristics or calibration status may lead to variations in beam‐axis stability. Third, in this study, beam‐axis deviations were analyzed with reference to the radiation dose center. However, the results might differ if the cone‐beam CT image isocenter or the mechanical isocenter, defined by a ball‐bearing phantom, were used as the reference point. Finally, in the present study, 10 MV and 10 FFF beams were not evaluated; however, the high–dose‐rate 10 FFF beam is clinically employed, for instance, in gated irradiation.^22^ Addressing these limitations in future studies will be important to establish the robustness and clinical utility of this method.

In conclusion, this study demonstrated that scintillation‐based imaging allows for high‐resolution, real‐time monitoring of beam‐axis stability. The consistently submillimeter deviations observed across different energies suggest the potential for this methodology to serve as a high‐precision QA tool. This method shows potential to complement conventional approaches by capturing angle‐dependent mechanical variations that are critical for treatments like cranial STI.

## AUTHOR CONTRIBUTIONS

All authors participated in the writing of this article and are responsible for its content. The authors declare that the contents of this manuscript have neither been published nor submitted for publication elsewhere.

## CONFLICT OF INTEREST STATEMENT

Shingo Ohira, Tenyoh Suzuki, and Keiichi Nakagawa belong is an endowment department, supported with an unrestricted grant from Elekta K. K. However, the sponsor had no role in this study.
